# A Tuning Fork Frequency Up-Conversion Energy Harvester

**DOI:** 10.3390/s21217285

**Published:** 2021-11-01

**Authors:** Qinghe Wu, Shiqiao Gao, Lei Jin, Xiyang Zhang, Zuozong Yin, Caifeng Wang

**Affiliations:** 1 State Key Laboratory of Explosion Science and Technology, Beijing Institute of Technology, Beijing 100081, China; 3120160078@bit.edu.cn (Q.W.); jinlei@bit.edu.cn (L.J.); yzz@bit.edu.cn (Z.Y.); 2 Beijing Institute of Remote Sensing Equipment, Beijing 100854, China; zhangxiyang2012@163.com; 3 Beijing Institute of Aerospace Control Device, Beijing 100039, China; rudygey@126.com

**Keywords:** tuning fork, vibration mode, piezoelectric, energy harvester, frequency

## Abstract

In this paper, a novel tuning fork structure for self-frequency up-conversion is proposed. The structure has an in-phase vibration mode and an anti-phase vibration mode. The in-phase vibration mode is used to sense the environment vibration, and the anti-phase vibration mode is used for energy conversion and power generation. The low-frequency energy collection and the high-frequency energy conversion can be achieved simultaneously. Theoretical and experimental results show that the tuning fork frequency up-conversion energy harvester has excellent performance. This structure provides the energy harvester with excellent output power in a low-frequency vibration environment. At the resonant frequency of 7.3 Hz under 0.7 g acceleration, the peak voltage is 41.8 V and the peak power is 8.74 mW. The tuning fork frequency up-conversion energy harvester causes the humidity sensor to work stably. The structure has the potential to power wireless sensor nodes or to be used as a small portable vibration storage device, especially suitable for the monitoring of the environment related to human movement.

## 1. Introduction

With the development of electronic technology, the energy demand of micro-power devices is decreasing, and it is possible to harvest energy from the environment to power these devices. [1,2]. Because vibrations have a high energy density [3,4,5], using vibration energy as the power source of energy harvesters is gaining favor. Kinetic harvesters mainly include the piezoelectric energy harvester [6,7,8,9,10,11,12], electromagnetic energy harvester [13,14,15,16,17], electrostatic energy harvester [18,19], friction energy harvester [20,21,22,23] and multi-mode composite energy harvester [24,25,26]. The piezoelectric energy harvester has the advantages of high output voltage, simple structure, easy integration, etc.; thus, it has received more attention [27].

Although considerable progress has been made in the research of piezoelectric energy harvesters over the past several decades, the traditional piezoelectric structure has a high natural frequency. Vibration in the environment is mostly of low frequency (0~30 Hz) [28,29,30]. When a traditional piezoelectric energy harvester is applied in a low-frequency environment, the output power is too low to operate stably with electrical appliances. This is mainly because the excitation frequency does not match the natural frequency of the energy harvester. Moreover, if the frequency is simply reduced, the power is limited even if the resonant state is reached. The reason for this power reduction is that low frequencies will increase internal resistance. Hence, the traditional piezoelectric structure greatly limits the effectiveness of the piezoelectric energy harvester in the low-frequency vibration environment.

In order to improve the output power and bandwidth of the energy harvester in the low-frequency environment, a frequency up-conversion energy harvester has been proposed [31,32,33,34,35,36]. Various frequency up-conversion (FUC) methods have been used [37,38,39,40,41], which can be divided into contact and non-contact methods. The frequency up-conversion technology can transform the low-frequency vibration in the environment into the high-frequency vibration needed by the energy harvester. When the FUC energy harvester is applied in a low-frequency environment, it can achieve a high-frequency vibration close to its natural frequency and improve the output power.

The contact method is realized by mechanical contact. Miah A. Halim et al. [42] proposed a frequency up-conversion piezoelectric vibration energy harvester driven by mechanical impact, employing a horizontally extended tip mass of a low-frequency driving beam to impact repeatedly on the free ends of two high-frequency unimorph-generating beams at the same time. Jinhui Zhang et al. [36]. proposed an impact-rope hybrid mechanism: the energy harvester comprises a high-frequency generating beam attached to a piezoelectric layer and a low-frequency driving beam. The two beams are placed face to face with a fixed space between them. They are connected mechanically at the tip using a rope with a certain margin.

The non-contact method is achieved through magnetic force. Tang et al. [43] introduced a non-contact energy harvester with frequency up-conversion based on magnetic repulsion. The harvester consists of a central sliding mass and a piezoelectric microcantilever beam, which uses a magnetic repulsion force to drive the bi-stable up-conversion of the high-frequency motor. Sliding mass magnets periodically repel each other, forcing the cantilever beam to bend and generate electricity. Wei Deng et al. [44] proposed a low-frequency electromagnetic energy harvester. The harvester converts rectilinear vibration to rotary motion using non-contact magnetic interaction, utilizing a multiple magnet/coil combination and magnetic potential wells which benefit from the rotary generator.

The traditional FUC systems require additional driver substructure, which is complicated and requires strict installation requirements. This also makes the structure of the energy harvester complex and difficult to miniaturize and integrate. To avoid this disadvantage, in this paper, a monomer frequency up-conversion structure is proposed. Compared with the traditional frequency up-conversion structure, this structure does not require additional frequency up-conversion substructure. The vibration modes of tuning fork structure are used to complete the frequency up-conversion function. The in-phase vibration mode of the structure is used to capture energy, while the anti-phase vibration mode is used to generate power. Frequency up-conversion is realized by single structure vibration mode. Only a simple tuning fork structure is needed to provide the energy harvester with the function of collecting low-frequency energy and high-frequency energy conversion at the same time.

Because no additional frequency up-conversion substructure is required, the energy harvester is simpler and easier to integrate. The power generation efficiency of the energy harvester is improved in the low-frequency vibration environment. Moreover, because the frequency of each order of the vibration mode of the structure increases with the increase in the order of the mode, the energy harvester is designed to use the low-order mode to capture energy and the high-order mode to convert energy for power generation, which is more in line with the needs of the energy harvester in the low-frequency vibration environment.

## 2. Design and Working Principle

### 2.1. The Structure Design

**Figure 1:**
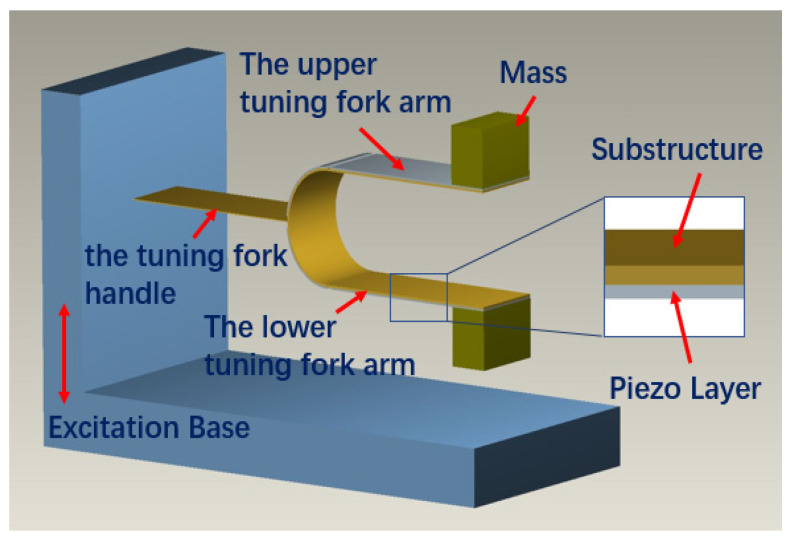
Structure of the tuning fork energy harvester.

In the tuning fork frequency up-conversion energy harvester, a tuning fork structure is used as the core component for energy conversion. The tuning fork energy harvester consists of two parts: a U-shaped structure with free ends (two connected tuning fork arms) and a tuning fork handle. The inner layer of the U-shaped structure is the substructure, which is made of beryllium bronze. The outer layer is PZT attached to the base beam. Each PZT is coated with a thin layer of silver electrode. The four piezoelectric patches are the parallel connection circuit. The two free ends of the arm are attached to the tip mass. The tuning fork handle is a short flexible sheet; one end is fixed at the center point of the U-shaped structure, and the other end is fixed to the base. Below the tuning fork is the fixed support of the tuning fork. It also acts as the surface of the impact when the tuning fork swings up and down. The geometric model is shown in Figure 1.

**Figure 2:**
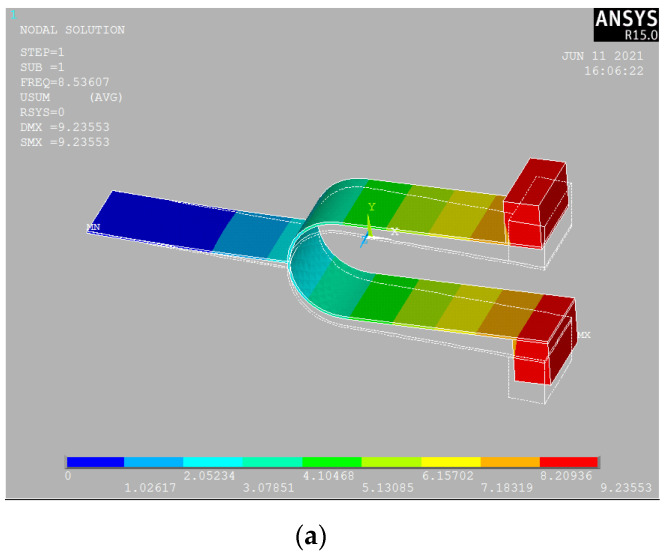

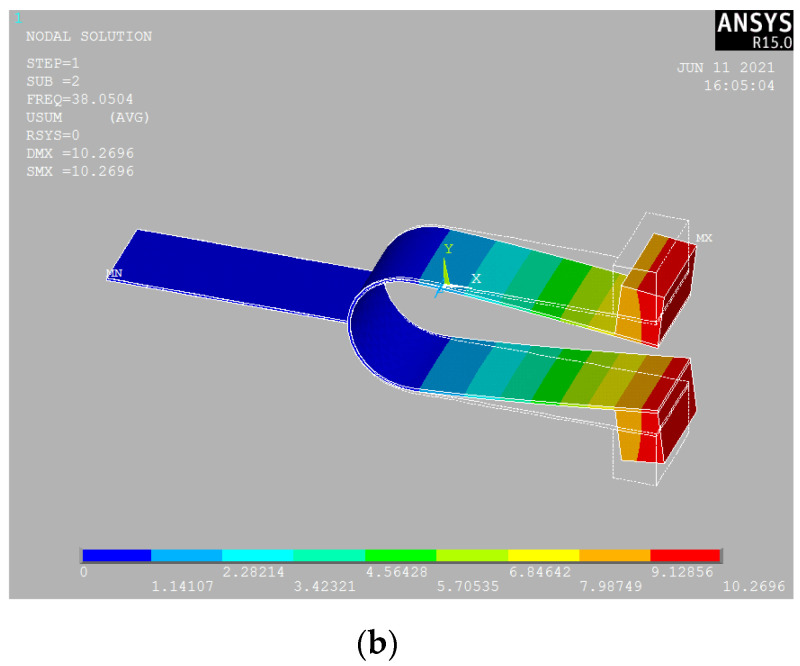
(**a**) The in-phase vibration mode and (**b**) the anti-phase vibration mode.

The tuning fork structure has two typical modes of vibration: one is the in-phase vibration mode of the tuning fork structure, and the other is the anti-phase vibration mode of the tuning fork structure. The in-phase vibration mode concerns the whole tuning fork system together with the tuning fork handle. The anti-phase vibration mode describes the closing or opening of the tuning fork arms, and the tuning fork handle is not involved. It is a high-frequency vibration mode. Due to the low stiffness of the tuning fork handle, the in-phase vibration mode is a low-frequency vibration mode. The frequency of the in-phase vibration mode is much lower than the anti-phase vibration mode. In the in-phase vibration mode, the two masses move in the same direction, as shown in Figure 2a. The symmetric vibration is the anti-phase vibration mode of the tuning fork, as shown in Figure 2b. The anti-phase vibration mode is the vibration of two masses around the midline.

### 2.2. Working Principle

**Figure 3:**
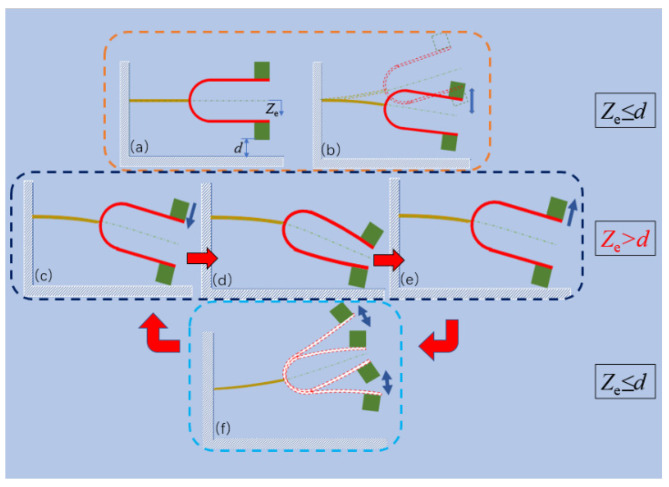
Motion location of the tuning fork energy harvester. (**a**) Stationary state, (**b**) small amplitude vibration, (**c**) impact, (**d**) the maximum deformation, (**e**) the moment of separation from the impact surface, and (**f**) the vibration of the tuning fork arm after it is separated from the impact surface.

The stationary state of the tuning fork, as shown in Figure 3a, where $z_{e}$ is the displacement of the tuning fork center line, and d is the impact gap.

The tuning fork vibrates when it is excited, and the displacement is small in the beginning, as shown in Figure 3b. As the displacement increases, the displacement is greater than the impact gap. The tip mass of the lower arm stops its downward motion temporarily after contacting the impact surface (Figure 3c). This is the in-phase vibration mode. The in-phase vibration mode is the lower-order mode of the tuning fork. The in-phase vibration mode is used to collect energy, such that the energy harvester has a low-frequency resonance with the environment in the process of capturing energy. More energy in the environment can thus be captured. After contacting the impact surface, the tip mass of the upper arm continues downward, and compresses the U-shaped structure to generate power, until achieving the maximum displacement, as shown in Figure 3d. Then, the tip mass of the upper arm begins to rise in the reverse direction. When the tip mass of the lower arm leaves the impact surface, as shown in Figure 3e, the tuning fork energy harvester moves upward until the displacement of the tuning fork center line reaches its highest point, then drops down, as shown in Figure 3f. At this point, the tuning fork completes one cycle of movement. Follow the above procedure, the tuning fork motion repeats the process and enters the next cycle. With the in-phase vibration mode, the two tip masses of the tuning fork always move towards or away from each other. This is the anti-phase vibration mode of the tuning fork. The anti-phase vibration mode is the higher-order vibration mode of the tuning fork. Because of the high-frequency vibration, the internal resistance of the energy harvester is low. In the anti-phase vibration mode, the PZT arranged in the high-frequency part completes the machine-electricity conversion, and thus energy in the environment is captured and converted into electrical output.

In this way, the tuning fork energy harvester avoids the disadvantages of high stiffness in energy capture and low stiffness in energy conversion, such that energy capture and power generation do not conflict with each other. This structure design improves the power of the energy harvester.

### 2.3. Electromechanical Coupling Dynamics Model

**Figure 4:**
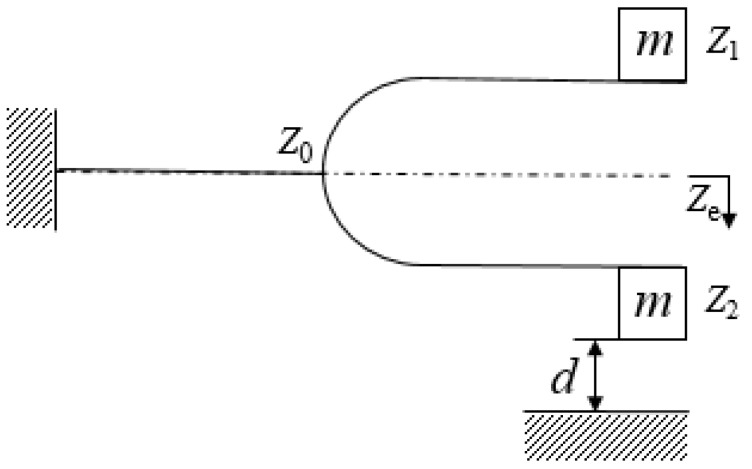
A schematic tuning fork structure.

Figure 4 shows the schematic tuning fork structure. When the displacement was greater than the impact gap ($z_{e}>d$), the analysis was performed in two statuses, the non-contact status and the high-frequency impact status.

#### 2.3.1. The Non-Contact Status

In this status, the dynamic equation of the tuning fork can be expressed as:

(1) $$\{\begin{array}{c} m{\ddot{z}}_{1}+c({\dot{z}}_{1}-{\dot{z}}_{0})+k(z_{1}-z_{0})+\theta V_{1}=f_{1}\\ m{\ddot{z}}_{2}+c({\dot{z}}_{2}-{\dot{z}}_{0})+k(z_{2}-z_{0})+\theta V_{2}=f_{2} \end{array}$$

where $z_{1}$ and $z_{2}$ are respectively the transverse displacement of the upper and lower tuning fork arm ends, $z_{0}$ is the transverse displacement of the connection between the tuning fork handle and the tuning fork arm, m is the mass of the end of the tuning fork arm, c is the damping coefficient of the tuning fork arm structure, k is the stiffness of the tuning fork arm, θ is the force–electric conversion coefficient of the piezoelectric patch on the tuning fork arm, $V_{1}$ and $V_{2}$ are respectively the voltages between the poles attached to the upper and lower tuning fork arms and $f_{1}$ and $f_{2}$ are the external force excitation of the upper and lower tuning fork arm end mass blocks, respectively.

By adding the two equations in set (1), we obtain:

(2) $$m({\ddot{z}}_{1}+{\ddot{z}}_{2})+c({\dot{z}}_{1}+{\dot{z}}_{2}-2{\dot{z}}_{0})+k(z_{1}+z_{2}-2z_{0})+\theta (V_{1}+V_{2})=f_{1}+f_{2}$$

According to the mechanical equilibrium conditions, the stiffness of the tuning fork handle and the tuning fork arm fulfil the following relationship:

(3) $$k_{0}z_{0}=k(z_{1}-z_{0})+k(z_{2}-z_{0})$$

where $k_{0}$ is the stiffness of the tuning fork handle structure, and the solution to the equation can be written as:

(4) $$z_{0}=\frac{k}{k_{0}+2k}(z_{2}+z_{1})$$

In the same way, the damping of the tuning fork handle and the tuning fork arm satisfies the following equation:

(5) $$c_{0}{\dot{z}}_{0}=c({\dot{z}}_{1}-{\dot{z}}_{0})+c({\dot{z}}_{2}-{\dot{z}}_{0})$$

where $c_{0}$ is the damping of the tuning fork handle, and ${\dot{z}}_{0}$ can be represented as:

(6) $${\dot{z}}_{0}=\frac{c}{c_{0}+2c}({\dot{z}}_{1}+{\dot{z}}_{2})$$

Substituting Equations (4) and (6) into Equation (2) leads to:

(7) $$m({\ddot{z}}_{1}+{\ddot{z}}_{2})+\frac{2cc_{0}}{c_{0}+2c}({\dot{z}}_{1}+{\dot{z}}_{2})+\frac{2kk_{0}}{k_{0}+2k}(z_{1}+z_{2})+\theta (V_{1}+V_{2})=f_{1}+f_{2}$$

The displacement of the tuning fork center line is:

(8) $$z_{e}=\frac{1}{2}(z_{1}+z_{2})$$

Equation (7) can be rewritten as:

(9) $$2m{\ddot{z}}_{e}+\frac{2cc_{0}}{c_{0}+2c}{\dot{z}}_{e}+\frac{2kk_{0}}{k_{0}+2k}z_{e}+\theta (V_{1}+V_{2})=f_{1}+f_{2}$$

Equation (9) is the in-phase vibration mode.

By subtracting the second equation from the first equation in Equation set (1), we obtain:

(10) $$m({\ddot{z}}_{1}-{\ddot{z}}_{2})+c({\dot{z}}_{1}-{\dot{z}}_{2})+k(z_{1}-z_{2})+\theta (V_{1}-V_{2})=f_{1}-f_{2}$$

The relative displacement between the two tip masses is:

(11) $$z_{h}=z_{1}-z_{2}$$

The displacement of one tip mass with respect to the center line is:

(12) $$z_{s}=\frac{1}{2}\left(z_{1}-z_{2}\right)$$

The corresponding relative velocity and relative acceleration are, respectively:

(13) $${\dot{z}}_{s}=\frac{1}{2}\left({\dot{z}}_{1}-{\dot{z}}_{2}\right)$$

and

(14) $${\ddot{z}}_{s}=\frac{1}{2}\left({\ddot{z}}_{1}-{\ddot{z}}_{2}\right)$$

thus

(15) $$2m{\ddot{z}}_{s}+2c{\dot{z}}_{s}+2kz_{s}+\theta (V_{1}-V_{2})=f_{1}-f_{2}$$

This is the anti-phase vibration mode.

When the tip mass of the tuning fork arm is excited by the environmental acceleration a(t), there are:

(16) $$f_{1}=f_{2}=ma(t)$$

Thus, the above equations of in-phase vibration modal and anti-phase vibration modal can be written as:

(17) $$\{\begin{array}{l} 2m{\ddot{z}}_{e}+\frac{2cc_{0}}{c_{0}+2c}{\dot{z}}_{e}+\frac{2kk_{0}}{k_{0}+2k}z_{e}+\theta (V_{1}+V_{2})=2ma(t)\\ 2m{\ddot{z}}_{s}+2c{\dot{z}}_{s}+2kz_{s}+\theta (V_{1}-V_{2})=0 \end{array}$$

The first equation in Equation (17) is the in-phase vibration mode, and the second equation is the anti-phase vibration mode. The mechanical–electrical conversion coefficient and voltage in the above equations are only related to the structure of the piezoelectric patch. When the piezoelectric patch is symmetrically attached to the outer side of the two tuning fork arms and the positive electrode of the voltage is set on the outer surface of the piezoelectric patch, $V_{2}=-V_{1}=-V$. The Equation set (17) can be rewritten as:

(18) $$\{\begin{array}{l} 2m{\ddot{z}}_{e}+\frac{2cc_{0}}{c_{0}+2c}{\dot{z}}_{e}+\frac{2kk_{0}}{k_{0}+2k}z_{e}=2ma(t)\\ m{\ddot{z}}_{s}+c{\dot{z}}_{s}+kz_{s}+\theta V=0 \end{array}$$

If $k_{0}$ is much smaller than k, the first equation above is the low-frequency forced vibration equation of the tuning fork in-phase mode, while the second equation is the high-frequency free vibration equation of the tuning fork anti-phase vibration mode. For low-frequency environmental excitation, a significant displacement response of the center line of the tuning fork can be obtained by the forced vibration of the low frequency, and the effective initial displacement of the high-frequency free vibration of the tuning fork is provided by the impact. The high-frequency free vibration of the anti-phase vibration mode of the tuning fork can obtain a continuous vibration with large amplitude, and the voltage output can be obtained through the force–electric conversion of the piezoelectric patch structure.

#### 2.3.2. The High-Frequency Impact Status

When the tip mass of the lower arm touches the bottom of the shell, it will collide, and the whole impact process contains two components. Two frequency components are involved in the impact. One component is the high-frequency impact between the tip mass of lower arm and the shell bottom, and the other is the low-frequency impact between the tuning fork structure and the shell bottom.

The high-frequency impact is mainly caused by the local elastic deformation of the tip mass of the lower arm during the impact, while the low-frequency impact is mainly caused by the elastic deformation of the tuning fork arm. In the cycle of a low-frequency impact, there will be many high-frequency impacts.

According to the Hertz contact mechanics theory, the impact force between the lower arm and the bottom of the shell can be roughly described as:

(19) $$f_{2}=-k_{m}\delta^{n}$$

where $k_{m}$ is the deformation stiffness of the tip mass, δ is the displacement of mass in the impact process, and n is a constant related to the shape of the mass structure.

The impact process of the tip mass of the lower arm is short. In this process, the effect of damping force and electromechanical coupling can be ignored, and the effect of the stiffness of the tuning fork handle can be ignored. The dynamic equation of the tuning fork arm tip mass can be written as:

(20) $$\{\begin{array}{l} m{\ddot{z}}_{1}+\frac{1}{2}k(z_{1}-\delta )+\theta (V_{1}-V_{2})=0\\ m\ddot{\delta }+\frac{1}{2}k(\delta -z_{1})=-k_{m}\delta^{n} \end{array}$$

where $z_{1}-\delta \approx z_{1}$. At the same time, the stiffness of the tuning fork arm k is much smaller than the tip mass of the lower arm $k_{m}$, and we can assume that the ratio is a small quantity $\varepsilon =\frac{k}{k_{m}}$; thus, Equation (20) can be reduced to:

(21) $$\{\begin{array}{l} m{\ddot{z}}_{1}+\frac{1}{2}kz_{1}+\theta (V_{1}-V_{2})=0\\ m\ddot{\delta }+\frac{1}{2}\varepsilon k_{m}(\delta -z_{1})=-k_{m}\delta^{n} \end{array}$$

The initial conditions are:

(22) $${\dot{z}}_{1}(t=0)=\dot{\delta }(t=0)=v_{0}$$

The multi-scale perturbation method is used to analyze and solve the problem. The first-order approximate solution is:

(23) $$\delta =\frac{v_{0}}{\omega_{m}}\cos\frac{1}{2}\frac{\omega^{2}}{\omega_{m}}t\sin\omega_{m}t-\frac{1}{2}\frac{\varepsilon \omega_{m}{}^{2}}{\omega_{m}{}^{2}-\omega^{2}}(\frac{v_{0}}{\omega_{m}}\cos\omega_{m}t-\frac{v_{0}}{\omega }\sin\omega t)$$

where $\varepsilon =\frac{k}{k_{m}}$. It is observed that if n=1, then its impact period is $\tau =\frac{2\pi }{\omega_{m}}$, where $\omega_{m}=\sqrt{\frac{k}{m}}$ is the frequency of high-frequency impacts.

## 3. Calculations and Experiment

**Figure 5:**
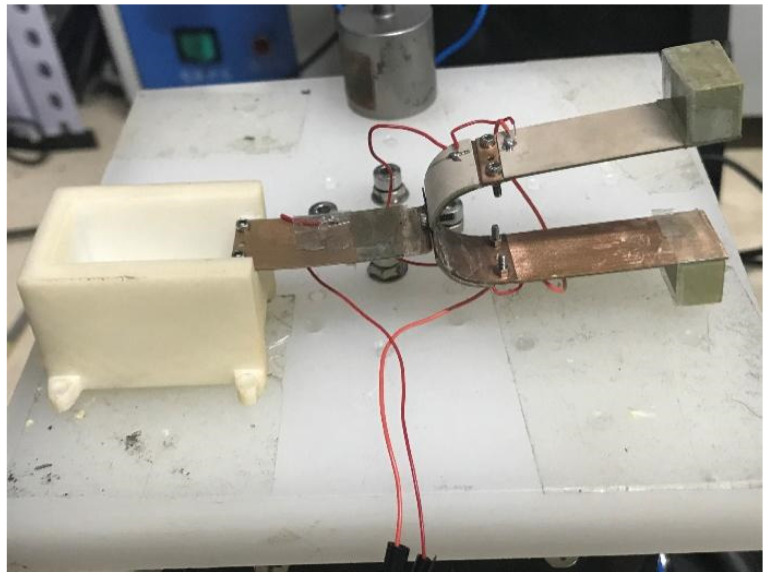
Photograph of the fabricated prototype.

The design and production were carried out according to Figure 1. A photograph of the fabricated prototype is shown in Figure 5. The parameters of the tuning fork energy harvester are shown in Table 1.

**Figure 6:**
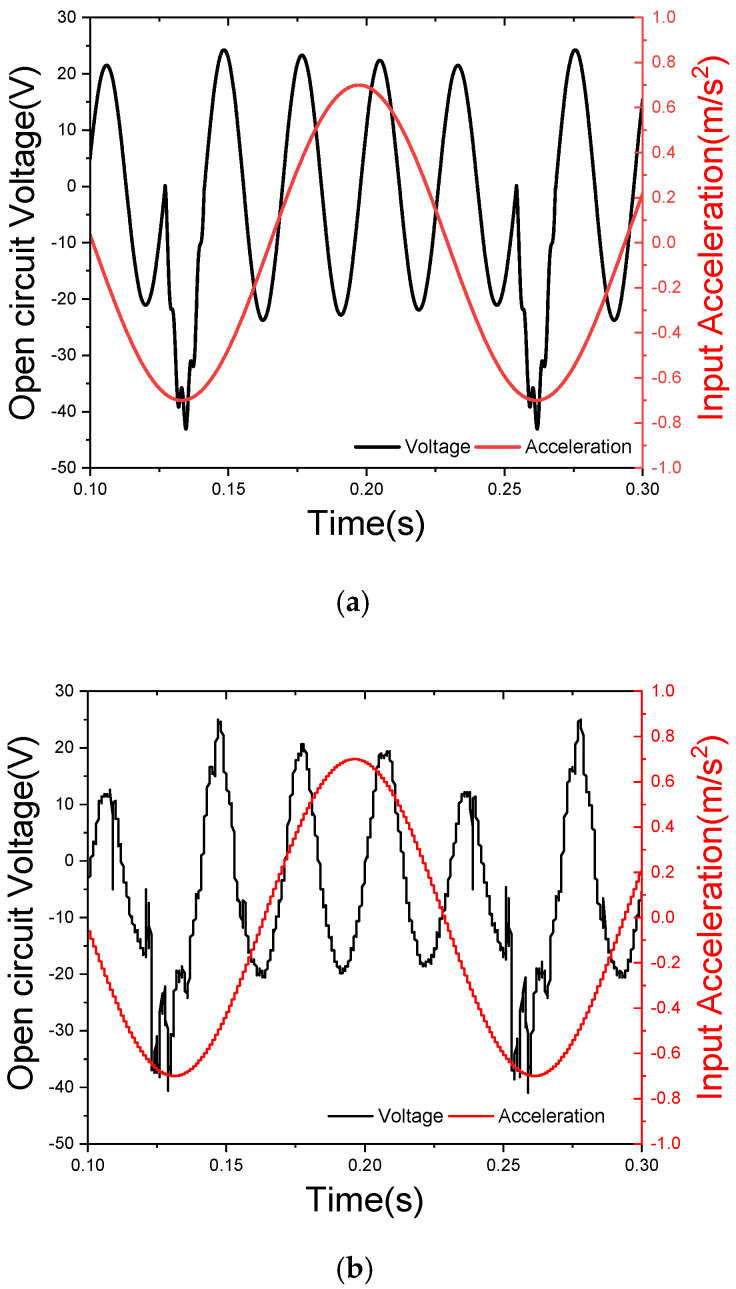
(**a**) The calculated open circuit voltage waveform and (**b**) the measured instantaneous voltage waveform.

According to the calculation of parameters in Table 1, under an excitation acceleration of 0.7 g, the initial impact distance between the free end of the lower arm of the tuning fork and the impact surface is 1.5 cm. The waveform of the voltage, changing with time, can be obtained from Equations (18) and (21), as shown in Figure 6a. During the impact period, as the free end of the lower arm stops suddenly, the impact results in a significant peak voltage. The energy harvesting frequency is 7.7 Hz and the power generation frequency is 34.78 Hz in the resonant state. In other words, the in-phase mode frequency is 7.7 Hz, and the anti-phase vibration mode frequency is 34.78 Hz. The peak voltage of the impact period is 44.54 V. The vibration attenuates freely four times and then enters the next impact period. The peak voltage of the separation period is 25.16 V.

Each vibration cycle consists of two stages, the impact stage and the separation stage, as shown in Figure 6a. The voltage amplitude after each impact is significantly higher than that in the free attenuation stage, and the voltage curve after the impact can be seen with high-frequency vibration, which is the result of the superposition of the waveform generated by the tip mass impact on the vibration curve of the tuning fork arm.

In order to study the performance of the tuning fork energy harvester in more detail, the experimental prototype of the energy harvester was fixed on the excitation platform for an experimental test.

**Figure 7:**
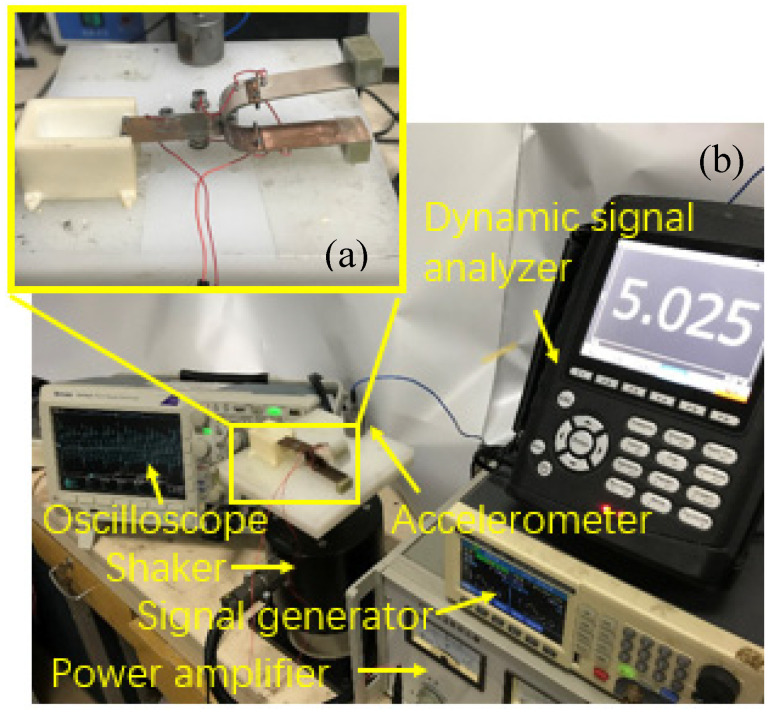
Experimental prototype and test system. (**a**) The physical picture of the experimental prototype; (**b**) tuning fork piezoelectric energy harvester and a complete test system.

Figure 7 shows the setup for testing the tuning fork energy harvester. The control signal of the experimental device is generated by the signal generator, amplified by the power amplifier, and then transmitted to the vibration exciter to control the vibration of the exciter. The harmonic excitation is used as the input signal of the system. The accelerometer is fixed on the excitation platform to monitor the input vibration of the energy harvester. The oscilloscope is connected to the energy harvester to measure and record the output characteristics of the energy harvester. In order to verify the relationship between the upper and lower arms of the tuning fork and their parallel connection, frequency sweep tests were carried out, respectively.

As shown in Figure 6b, the experimental test results show that the energy harvesting frequency is 7.3 Hz and the power generation frequency is 34.1 Hz in the resonant state. In other words, the in-phase mode frequency is 7.3 Hz, and the anti-phase vibration mode frequency is 34.1 Hz. The peak voltage of the impact period is 41.7 V. The peak voltage of the separation period is 25.6 V. These results are in agreement with the theoretical calculation, which shows that the impact period has obvious high-frequency vibration characteristics. During the separate period, the tuning fork is in free vibration. The separate period vibration output voltage decays exponentially with time. The vibration attenuates freely four times and then enters the next impact period. The voltage amplitude decays slowly; thus, the tuning fork energy harvester is able to maintain a high voltage peak while operating. In the free attenuation generation period, the stronger stage is retained, while the weaker stage is cut off by the next impact and directly enters the next impact period. Significantly reducing the proportion of low energy output time maintains a high output power. It is beneficial to improve the power of the energy harvester and the output stability.

The comparison between the theoretical and experimental results shows that the experimental results coincide with the theoretical design. The correctness of the theoretical model is verified.

**Figure 8:**
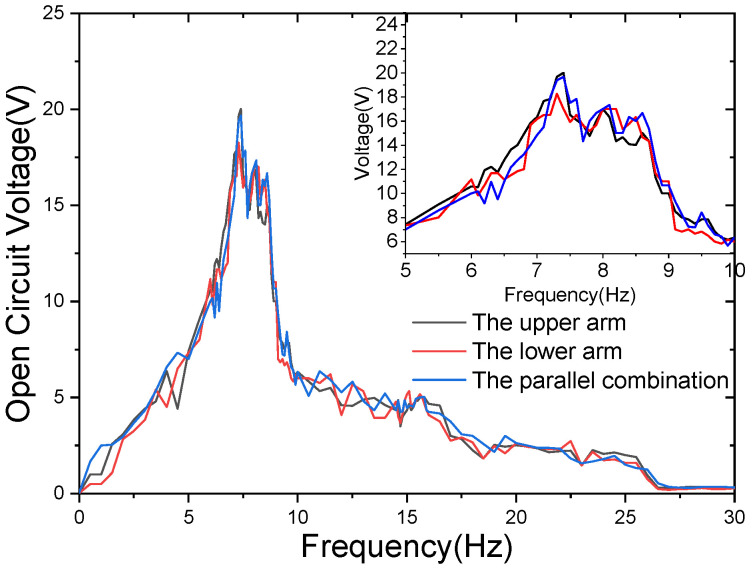
Tuning fork upper arm, lower arm, and double arm parallel sweep voltage, under an acceleration of 0.7 g, with impact spacing of 1.5 cm.

For an acceleration of 0.7 g and an impact gap of 1.5 cm, the experimental results are shown in Figure 8. It can be seen that the voltage difference between the upper arm, the lower arm, and the paralleling of the tuning fork is small. It is also verified that the parallel connection of the piezoelectric patch conforms to the parallel connection of the power supply.

Although the lower arm of the tuning fork collides with the impact surface and the upper arm of the tuning fork does not, the peak voltage of the two arms of the tuning fork does not show an obvious difference under the various excitation frequencies upon the impact surface.

**Figure 9:**
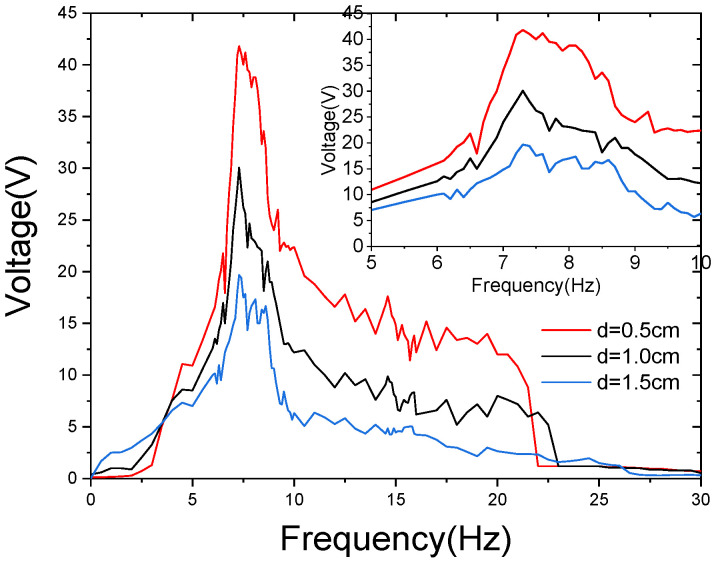
The measured open-circuit voltages against operating frequencies, ranging from 0 to 30 Hz, under various impact gaps.

Figure 9 shows the sweeping voltage of the energy harvester with 0.7 g acceleration and different impact gaps. The peak voltage of the energy harvester is in the range of 5–10 Hz. Several small amplitude fluctuations occur on this peak. The output peak decreases slowly in the 10–22 Hz range. At a level greater than 22 Hz, the voltage drops significantly. When the impact gap d=1.5 cm, the peak voltage is 19.68 V. With the exception that the energy harvester is better than d=1.0 cm and d=1.5 cm in the range less than 3.5 Hz, all other frequency ranges are smaller than the other two cases. When the impact gap d=1.0 cm, the peak voltage is 30.07 V. When the impact gap d=1.5 cm, the peak voltage is 41.9 V.

Therefore, when the impact gap is in the range of 0.5–1.5 cm, the size of the impact gap has little effect on the resonant frequency of the energy harvester. The increase in the impact gap will allow the energy harvester to obtain a relatively higher voltage at a lower frequency, while the decrease in impact gap will allow the energy harvester to obtain a relatively higher peak voltage.

**Figure 10:**
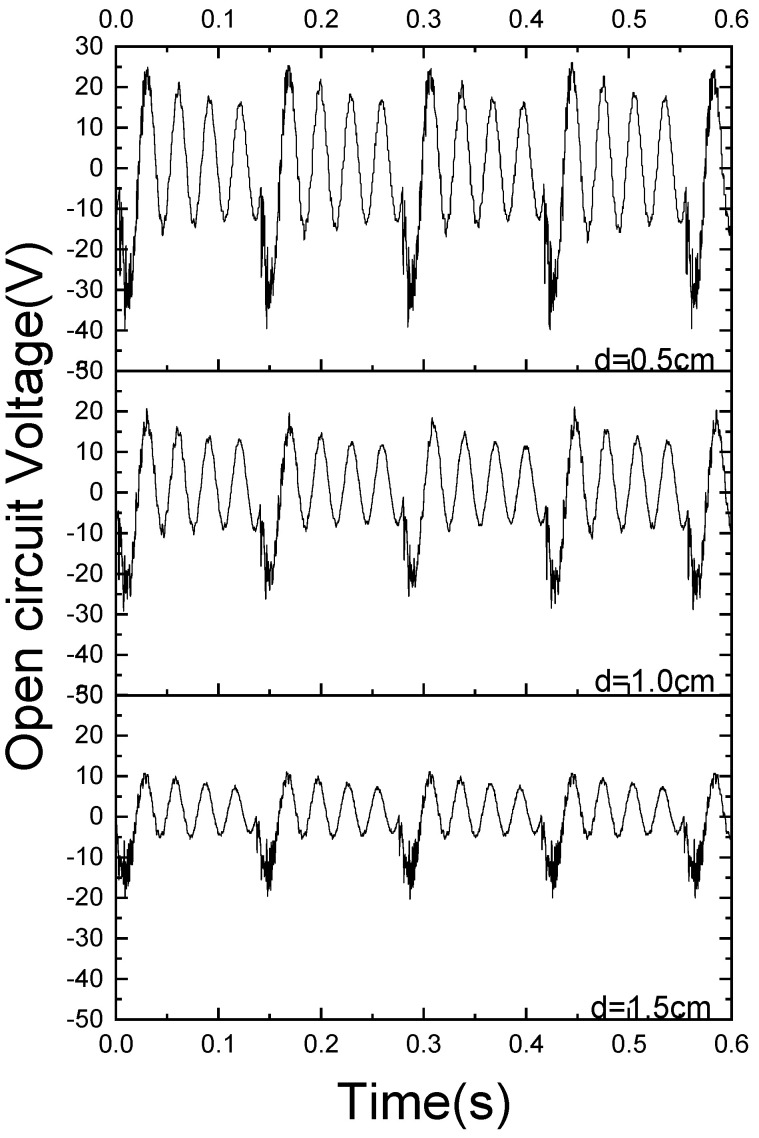
The instantaneous open-circuit voltage of the tuning fork energy harvester with different impact spacing under an acceleration of 0.7 g.

Figure 10 shows the time variation curve of the open-circuit voltage corresponding to an excitation acceleration of 0.7 g and varying impact gap d. When the impact spacing is within the range of 0.5–1.5 cm, three different impact gap experiments are basically identical, except for some differences in voltage amplitude and high-frequency impacts.

As acceleration increases, the voltage value increases. The peak power delivered to the load is [35]:

(24) $$P=\frac{V^{2}R_{L}}{{\left(R_{L}+R_{S}\right)}^{2}}$$

where P is the power, *V* is the voltage, $R_{L}$ is the load resistance, and $R_{S}$ is the internal resistance of the energy harvester. If $R_{L}=R_{S}$, P is at its maximum. The output power is maximum when the external load resistance is equal to the internal resistance.

**Figure 11:**
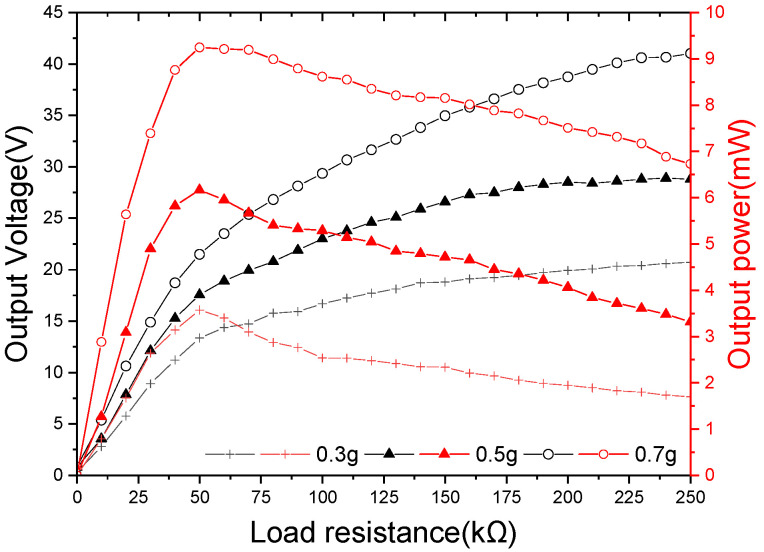
The peak output voltages (dashed lines) and peak output powers (solid lines) against various load resistances at the resonant frequency of 7.7 Hz under varying base acceleration.

Figure 11 shows the peak voltage and peak powers delivered to the load. The optimum load resistance for maximum power transfer was determined by varying the load resistance values at 7.7 Hz under various accelerations. It was found from the voltage curve changing with load resistance that the voltage across the load increases as the load resistance increases. The peak output power is delivered when the load resistance matches the source resistance, that is, the external load resistance is equal to the source resistance. In the experiments, a maximum peak power of 8.74 mW was delivered to a 50 kΩ resistor.

## 4. Power Supply Experiment for Electrical Appliances

**Figure 12:**
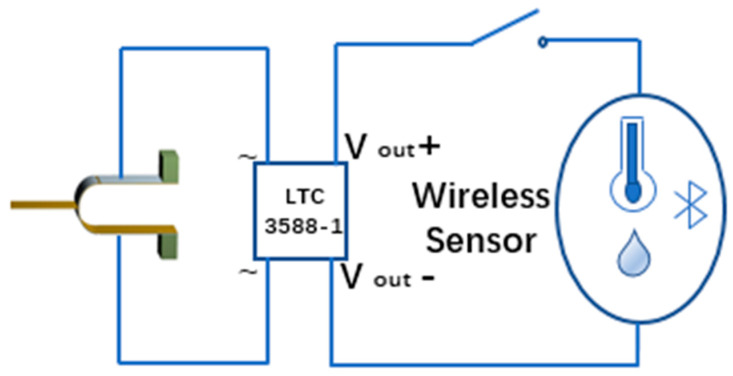
Circuit diagram used to power humidity sensors.

To demonstrate a practical application, a humidity sensor was connected to the circuit and powered by an energy collector. After connecting the tuning fork energy harvester to the power management chip LTC3588, it was connected to the humidity sensor for a power supply experiment test, as shown in Figure 12.

**Figure 13:**
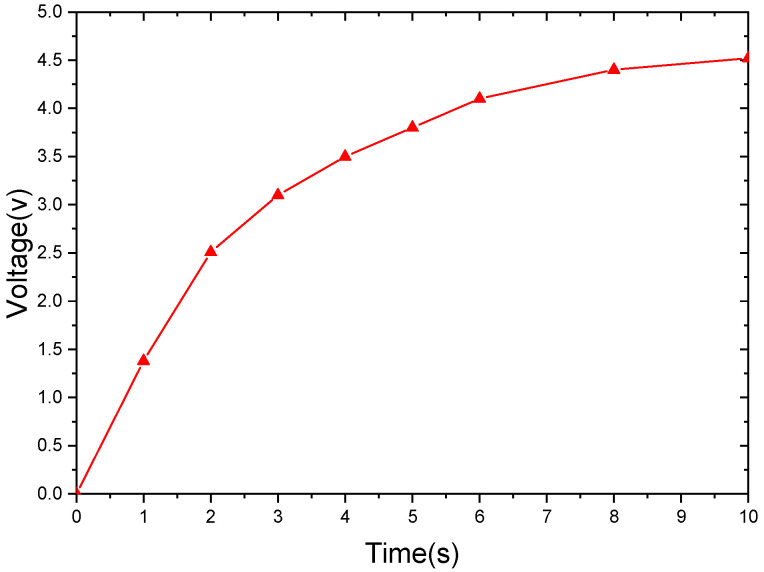
Charging behavior using LTC3588 (a=0.7 g,d=1.5 cm).

The excitation acceleration is 0.7 g, the impact spacing is 1.5 cm, and the excitation frequency is 7.7 Hz. The experimental results show that the voltage starts to rise gradually because the capacitor in LTC3588 needs to be charged for a short time after the excitation is applied and then becomes stable after about 8 s, as shown in Figure 13.

**Figure 14:**
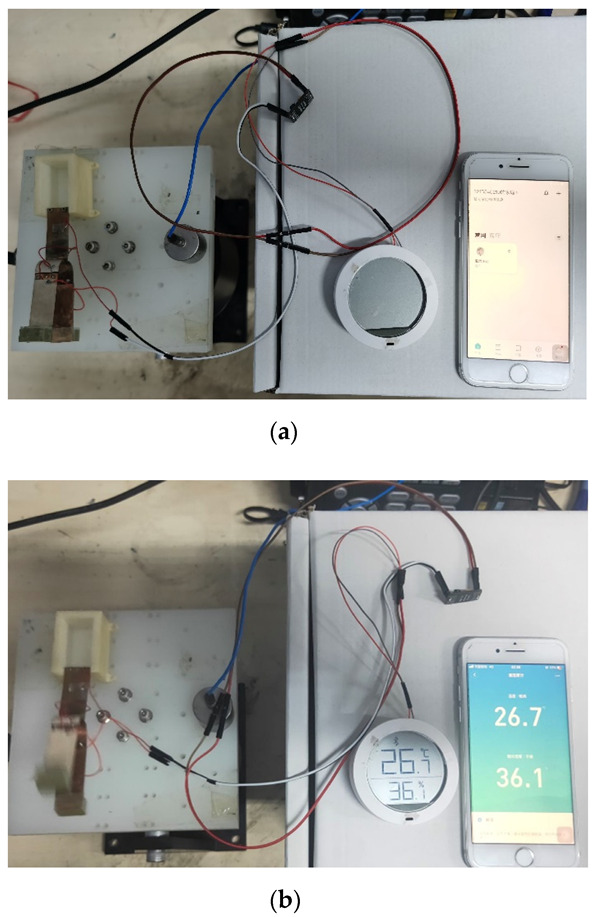
Pedometer powered by the tuning fork energy harvester. (**a**) The tuning fork energy harvester not working; (**b**) the tuning fork energy harvester working.

After connecting, as shown in Figure 14a, the tuning fork energy harvester can produce a stable output and cause the humidity sensors to work, as shown in Figure 14b.

## 5. Conclusions

In this paper, a frequency up-conversion energy harvester based on the modal of a single structure is proposed. The different modes of a single structure are used to realize energy capture and energy conversion, and the function of frequency up-conversion is realized. It not only solves the problem of the high frequency of the piezoelectric patch being unable to match the low-frequency environment vibration, but it is also different from the traditional frequency up-conversion method, which does not require an additional frequency conversion mechanism, simplifying the structure and reducing the installation difficulty.

Through theoretical calculation and experimental verification, it is proven that the structure can realize the frequency up-conversion function through its own mode. The results of theoretical calculations and the experimental test are in good agreement with each other. When the impact spacing increases in the range of 0–1.5 cm, the power of the energy harvester increases, and the resonant frequency hardly changes. When the acceleration is 0.7 g and the impact spacing is 1.5 cm, the peak open-circuit voltage is 41.8 and the peak power is 8.74 mW.

Each impact can obtain a peak voltage, and then the voltage enters a state of free decay. The high-output part of the early attenuation phase is retained, the low-output phase is truncated by the next impact, and the truncated part is replaced by the high-output stage generated by the next impact. In this way, the high energy output stage is more compact, the low power output stage is significantly reduced, and the working time possesses high power, which greatly improves the output power of the energy harvester.

The power management chip LTC-3588 was used to rectify and filter the output of the tuning fork energy harvester and the power supply test for the humidity sensors. The experimental results show that the tuning fork energy harvester has the ability to provide stable electric energy for humidity sensors, which allow them to work normally. It can be used as a stable source of power for small electrical appliances.

The device has the potential to be used in low-frequency vibration environments and can provide power for wearable devices or serve as a self-charging charger.

## Figures and Tables

**Table 1:** Material properties and structural parameters of the proposed energy harvester.

Parameter	Value
Transverse piezoelectric constant (*d*_31_)	−273 pm/V
Young’s modulus of stainless steel (*Y_s_*)	130 GPa
Young’s modulus of PZT unimorph (*Y_p_*)	56 GPa
Dielectric constant of PZT unimorph (*ε*_33_)	1.389 × 10^−8^ F/m
Damping ratio of system (*ζ*)	0.02
Quality of proof mass1 (*m*)	10 *g*
Size of the handle (l_h_*b_h_*h_h_)	50 mm × 15 mm × 0.2 mm
Size of the straight part (l_s_*b_s_)	40 mm × 16 mm
Radius of the arc (*R*) Thickness of base beam (*h_b_*)	10 mm 0.2 mm
Size of PZT unimorph (l_p_*b_p_*h_p_)	20 mm × 16 mm × 0.3 mm

## Data Availability

The data that support the findings of this study are available from the corresponding author upon reasonable request.
